# Healthcare benefits linked with Below Poverty Line registration in India: Observations from Maharashtra Anaemia Study (MAS)

**DOI:** 10.12688/f1000research.10556.1

**Published:** 2017-01-09

**Authors:** Anand Ahankari, Andrew Fogarty, Laila Tata, Puja Myles

**Affiliations:** 1 Division of Epidemiology and Public Health, University of Nottingham, Nottingham, UK; 2 Halo Medical Foundation, Maharashtra, India

**Keywords:** India, Below Poverty Line, Healthcare benefits, Maharashtra Anaemia Study

## Abstract

A 2015 *Lancet* paper by Patel *et al.* on healthcare access in India comprehensively discussed national health programmes where some benefits are linked with the country’s Below Poverty Line (BPL) registration scheme. BPL registration aims to support poor families by providing free/subsidised healthcare. Technical issues in obtaining BPL registration by poor families have been previously reported in the Indian literature; however there are no data on family assets of BPL registrants. Here, we provide evidence of family-level assets among BPL registration holders (and non-BPL households) using original research data from the Maharashtra Anaemia Study (MAS).

Social and health data from 287 pregnant women and 891 adolescent girls (representing 1178 family households) across 34 villages in Maharashtra state, India, were analysed. Several assets were shown to be similarly distributed between BPL and non-BPL households; a large proportion of families who would probably be eligible were not registered, whereas BPL-registered families often had significant assets that should not make them eligible. This is likely to be the first published evidence where asset distribution such as agricultural land, housing structures and livestock are compared between BPL and non-BPL households in a rural population. These findings may help planning BPL administration to allocate health benefits equitably, which is an integral part of national health programmes.

## Introduction

Patel *et al.* (2015) provided a comprehensive picture of the current Indian healthcare structure, and also mentioned the National Health Mission’s (NHM) initiative to target inequalities in healthcare access ^1^. Such national health programmes use the ‘Below Poverty Line’ (BPL) registration status to identify deprived families and provide them with free/subsidised healthcare services ^2^. The registration is allocated at family level, based on a scoring system calculated using family level assets such as agricultural land, housing structures, electricity supplies, household equipment. The scoring system varies within Indian states. The BPL status provides access to free healthcare facilities along with monthly access to subsidised food products including but not limited to wheat, rice, cooking oil and sugar.

There are no data on family assets of BPL registrants. Therefore, in this study, we provide evidence of family-level assets among BPL registration holders (and non-BPL households) using research data we collected previously for the Maharashtra Anaemia Study (MAS) ^3– 5^. The MAS was conducted though a joint collaboration of Halo Medical Foundation (HMF), India and the University of Nottingham, UK.

## Methods

The MAS was conducted to identify risk factors associated with anaemia in pregnant women (3 to 5 months gestation), and in 13 to 17 year old adolescent girls, living in 34 villages of the Osmanabad district of Maharashtra state of India. MAS collected information on health and social conditions along with blood investigations to examine anaemia risks in rural Indian communities. Additional details of the MAS project are published elsewhere ^3– 5^.

Data collection also included information on family assets such as agricultural land, housing structure, livestock, automobiles, employment, and home electronics. In this research note, we evaluated family level assets in relation with the BPL registration. The comparison was made in BPL and non-BPL holders for each asset using Chi-square statistics in Stata Software (V.13.1, Texas, USA). 

In total, 287 pregnant women and 1010 adolescent girls participated in data collection, giving an overall response rate of 95%. We selected one person per household at random for the analysis, which resulted in 287 pregnant women ( Dataset 1 ^6^), all from unique households, and 891 adolescent girls ( Dataset 2 ^7^). Therefore, 1178 total households across 34 villages (a population of approximately 65,500) were used in analyses. Written approval was obtained from each study participant and their guardian prior to data collection, and the same was counter signed by the primary investigator (AA). The study was approved by the Institutional Ethics Committee of Government Medical College of Aurangabad, India (Reference number: Pharma/IEC/GMA/196/2014), and also by the Nottingham University Medical School Research Ethics Committee (Reference number: E10102013).

Pregnant Women MAS Project The data has 287 pregnant women participants with self-explanatory variables on BPL registration, and related assets analysed in the paper. Click here for additional data file. Copyright: © 2017 Ahankari A et al. 2017 Data associated with the article are available under the terms of the Creative Commons Zero "No rights reserved" data waiver (CC0 1.0 Public domain dedication).

Adolescent Girls MAS Project The data has 891 adolescent girls participants with self-explanatory variables on BPL registration, and related assets analysed in the paper. Click here for additional data file. Copyright: © 2017 Ahankari A et al. 2017 Data associated with the article are available under the terms of the Creative Commons Zero "No rights reserved" data waiver (CC0 1.0 Public domain dedication).

## Results

36.4% of adolescent girls (325/891), and 37.6% (108/287) pregnant women in our study had current BPL registration. 32.3% (105/325) of adolescent girl families with BPL registration had more than 5 acres of farming land, and 54.4% (177/325) had a colour television. Overall, of the 6 assets we assessed, 3 showed no significant differences in distribution (p>0.05) between BPL registered and non-registered families of adolescent girls ( Table 1).

**Table 1.** Distribution of family assets in non-BPL and BPL registrants observed among adolescent girls and pregnant women participants living in Osmanabad district of Maharashtra, India.

I) Adolescent girls [N=891, Non-BPL registrants 566 (63.5%), and BPL registrants 325 (36.4%)]
Below Poverty Registration	Non-BPL (percentage)	BPL registrants (percentage)	P value
**Asset 1: Farming land**
a. No farming land	250 (44.1%)	69 (21.2%)	<0.001
b. ≤ 5 acres of land	222 (39.2%)	151 (46.4%)
c. > 5 acres of land	94 (16.6%)	105 (32.3%)+
**Asset 2: Livestock**
a. Without any livestock	160 (28.2%)	102 (31.3%)	0.32
b. Holds livestock	406 (71.7%)	223 (68.6%)
**Asset 3: House structure**
a. Participants with temporary house	2 (0.3%)*	2 (0.6%)	0.34
b. Participants with semi-permanent house	487 (86.0%)	289 (88.9%)
c. Participants with permanent house	77 (13.6%)	34 (10.4%)+
**Asset 4: Television**
a. No	213 (37.6%)	148 (45.5%)	0.02
b. Yes	353 (62.3%)	177 (54.4%)+
**Asset 5: At least one mobile phone in** **the family**
a. No	27 (4.7%)	28 (8.6%)	0.02
b. Yes	539 (95.2%)	297 (91.3%)
**Participant temporally employed such** **as farm based labour work**
a. Not employed	526 (92.9%)	298 (91.6%)+	0.49
b. Temporarily employed	40 (7.0%)	27 (8.3%)
II) Pregnant women [N=287, Non-BPL registrants 179 (62.4%) and BPL registrants 108 (37.6%)]
Below Poverty Registration	Non-BPL (percentage)	BPL registrants (percentage)	P value
**Asset 1: Annual Income**
a. Less than 50,000/- INR (500 GBP)	76 (42.4%)*	61 (56.4%)	0.006
b. Between 50,001 to 100,000/-INR (501-1000 GBP)	84 (46.9%)	45 (41.6%)+
c. Above 100,001/- INR (1001 GBP and above)	19 (10.7%)	2 (2%)+
**Asset 2: Farming Land**
a. No farming land	30 (16.7%)	39 (36.1%)	<0.001
b. ≤ 5 acres of land	50 (27.9%)	39 (36.1%)
c. > 5 acres of land	99 (55.4%)	30 (27.8%)+
**Asset 3: Water motor pump at farm**
a. No	74 (41.3%)	76 (70.3%)	<0.001
b. Yes	105 (58.7%)	32 (29.7%)+
**Asset 4: Livestock**
a. Without any livestock	45 (25.1%)	33 (30.5%)	0.31
b. Holds livestock	134 (74.9%)	75 (69.5%)
**Asset 5: House structure**
a. Participants with temporary house	1 (0.5%)*	1 (0.9%)	0.22
b. Participants with semi-permanent house	162 (90.5%)	103 (95.3%)
c. Participants with permanent house	16 (9%)	4 (3.8%)+
**Asset 6: Family owns a three/four** **wheeler vehicle or any agricultural** **vehicle**
a. No	151 (84.3%)	99 (91.6%)	0.07
b. Yes	28 (15.7%)	9 (8.4%)+
**Asset 7: Family owns a two wheeler**
a. No	110 (61.4%)	71 (65.7%)	0.46
b. Yes	69 (38.6%)	37 (34.3%)+
**Asset 8: Television**
a. No	50 (27.9%)	40 (37%)	0.10
b. Yes	129 (72.1%)	68 (63%)+
**Asset 9: At least one mobile phone in** **the family**
a. No	5 (2.7%)	5 (4.6%)	0.41
b. Yes	174 (97.3%)	103 (95.4%)
**Any assets sold in last 12 months (such** **as land, livestock, agricultural tools/** **equipment, house vehicle, gold or any** **other valuable items)**
a. No	149 (83.3%)	89 (82.5%)	0.85
b. Yes	30 (16.7%)	19 (17.5%)

+: Those who are likely to be ineligible but hold BPL registration. *: Those who appeared to be eligible but did not have registration. Annual income is also presented in Great Britain Pound (GBP) based on the conversion rate of 1 GBP= 100 Indian Rupees (INR). Note: Family income/assets was defined as an immediate family’s resources only. For example: for adolescent girls, it included participants’ parents’ (mother and father only) income/assets; among pregnant women, it included participants’ (pregnant woman) and husbands’ income/assets only. P values were calculated using chi square test.

Among families of pregnant women, 6 out of 9 assets assessed showed no significant differences (p>0.05) between BPL registered and non-registered. Furthermore, 2% of the families of BPL registrants (2/108) had an annual income greater than 100,000 INR (~1000 GBP), 27.8% had more than 5 acres of land (30/108), and 8.4% had three/four wheeler vehicles (9/108).

## Discussion

Non-eligible families holding the BPL registration are likely to increase burden on healthcare services, while those with greatest need may remain untreated due to absence of BPL registration, or inability to pay for healthcare services out of their own pockets ^2, 8^. Subsidising non-eligible BPL holders also increases the burden on government finances, which in light of the current fragile economic situation, is an important issue to address ^8^.

We observed several participants from both study groups in the MAS, who appeared eligible for the BPL scheme, but had not obtained the registration. Many participants reported technical difficulties as the reason for not having BPL registration. Some of these technical difficulties included having problems procuring the required documents from government officials, and being unable to complete paperwork and other legal documents that are needed to submit the BPL application. This suggests a need to re-evaluate and strengthen the current BPL registration system, and also demands further monitoring to ensure that poor families in need receive vital healthcare and other subsidy benefits. The National Health Mission’s initiatives are well meant and have the potential to provide universal health coverage in India; however, implementation is challenging. Strengthening the current BPL registration system and improving identification of poor and needy families might help with achieving the universal health model. This may also help in revising the current health budget to allocate funds for the improvement of the governmental health system. We welcome the review from Patel *et al*. (2015) and suggest continuing evaluation of both national health projects and the BPL registration process, which will be useful in underpinning healthcare facilities whilst widening access.

## Data availability

The data referenced by this article are under copyright with the following copyright statement: Copyright: © 2017 Ahankari A et al.

Data associated with the article are available under the terms of the Creative Commons Zero "No rights reserved" data waiver (CC0 1.0 Public domain dedication). 

**Dataset 1: Pregnant Women MAS Project.** The data has 287 pregnant women participants with self-explanatory variables on BPL registration, and related assets analysed in the paper.

doi, 10.5256/f1000research.10556.d148743 ^6^

**Dataset 2: Adolescent Girls MAS Project.** The data has 891 adolescent girls participants with self-explanatory variables on BPL registration, and related assets analysed in the paper

doi, 10.5256/f1000research.10556.d148744 ^7^

## Ethics statement

The study was approved by the Institutional Ethics Committee of Government Medical College of Aurangabad, India (Reference number: Pharma/IEC/GMA/196/2014), and also by the Nottingham University Medical School Research Ethics Committee (Reference number: E10102013). All participants and their guardians provided signed informed consent for the survey and blood withdrawal separately. Each consent was countersigned by the primary investigator (AA). Other than those who declined to participate, all adolescent girls and pregnant women received a standardised health report including information on their haemoglobin level and anaemia status along with facilitated access to educational materials on anaemia through the health NGO, Halo Medical Foundation’s (HMF) village based services. Participant health reports were also provided to the village health worker/government nurse with arrangements for free consultation and assistance if any significant health problems requiring further assessment or treatment were identified during the study. HMF’s hospital was also made available for free consultation as a primary referral centre if more specialist assessment or treatment was needed. On completion of data collection, an additional reminder letter was issued to village health workers indicating details of each severe anaemic case in their village to ensure that necessary medical advice and treatment was available.
