# Supplementary material for: Healthcare benefits linked with Below Poverty Line registration in India: Observations from Maharashtra Anaemia Study (MAS)
Source: F1000Res. 2017 Jan 9;6:25. [Version 1] doi: 10.12688/f1000research.10556.1 (PMC5302144; doi:10.12688/f1000research.10556.1)
Supplement: Peer review history

# Peer review: Referee response for version 1

**DOI:** 10.5256/f1000research.11375.r19814

**Published:** 2017-02-06

**Authors:** Umesh Wadgave, Mahesh Khairnar

**Affiliations:** 1 Bharati Vidyapeeth Deemed University Dental College and Hospital, Sangli, Maharashtra, India

It’s an interesting and valid research tapping the vital topic of distribution of BPL cards in India. Overall methodology of the research is sound.

 There are certain issues which require clarification from authors

1. The assets considered for issuing BPL cards in Maharashtra state are quite different from assets considered in the present study ( For details follow this link http://mahafood.gov.in/website/english/PDS.aspx). These differences should be considered before making recommendations about policy change in BPL card distribution.
2. Authors state that “ *Overall, of the 6 assets we assessed, 3 showed no significant differences in distribution (p>0.05) between BPL registered and non-registered families of adolescent girls (Table 1)*.” But all the three assets which didn’t show significant difference are not listed in criteria to issue BPL Cards as per Maharashtra State.
3. Table 1 shows that about 44% of Non-BPL holders don’t have farming land which is far more than BPL card holders where only 21% don’t have farming land. However, in Table 2 it’s just opposite where 16.7% Non-BPL holders and 36.1 % BPL holders don’t have farming land. This contradicting finding needs to be justified in the discussion.
4. Why different assets were considered for adolescent girls (5 assets) and pregnant women (9 assets).
5. What is the necessity to stratify the analysis for adolescent girls and pregnant women separately? Authors could have clubbed the data and did the analysis which will increase the power of the study.
6. The sample for the present study consists of households having adolescent girls and pregnant women which will limit the generalizability of the study findings. Hence, the issue of generalizability of study findings should be discussed.
7. In the discussion, it is necessary to discuss study limitations and future scope of doing research on this topic.

We have read this submission. We believe that we have an appropriate level of expertise to confirm that it is of an acceptable scientific standard.

# Peer review: Referee response for version 1

**DOI:** 10.5256/f1000research.11375.r19107

**Published:** 2017-01-19

**Authors:** Sunil M. Sagare

**Affiliations:** 1 Department of Community Medicine, MIMSR Medical College, Latur, Maharashtra, India

Authors had given importance of subject appropriately, clarified ethical considerations, self-explanatory results and well discussion on the issue.  

 The authors pinpointed the below poverty line (BPL) issue in the form of non-eligible BPL registrants and eligible BPL non-registrants. The article raise the following questions:

1. Undue healthcare advantage taken by non-eligible BPL registrants
2. Disadvantage to needy eligible BPL families due to their non-registration.

Suggestions given for above mentioned questions are very much relevant and if implemented will help to reduce burden on government and healthcare finances as well.

I have read this submission. I believe that I have an appropriate level of expertise to confirm that it is of an acceptable scientific standard.
